# Comparison of ultrasound-guided modified thoracoabdominal nerve block through perichondrial approach (M-TAPA) and lateral transversus abdominis plane block on postoperative pain and quality of recovery after laparoscopic inguinal hernia repair: a prospective observational comparative study

**DOI:** 10.1186/s12871-026-04170-7

**Published:** 2026-08-13

**Authors:** İnan Sarıdede, Öznur Demiroluk, Doğa Meriç Yükselen, Birol Ağca, Arzu Yıldırım Ar

**Affiliations:** 1 Department of Anesthesiology and Reanimation, Fatih Sultan Mehmet Health Application and Research Center, University of Health Sciences, İçerenköy, D100, Hastane St., 34752 Ataşehir, Istanbul, Türkiye; 2 Department of General Surgery, Fatih Sultan Mehmet Health Application and Research Center, University of Health Sciences, İçerenköy, D100, Hastane St., 34752 Ataşehir, Istanbul, Türkiye

**Keywords:** Modified thoracoabdominal nerve block, M-TAPA, Transversus abdominis plane block, Laparoscopic inguinal hernia repair, Postoperative pain, Quality of recovery, Regional anesthesia

## Abstract

**Background:**

The modified thoracoabdominal nerve block through perichondrial approach (M-TAPA) and the lateral transversus abdominis plane (TAP) block are both used within multimodal analgesia after abdominal surgery, but comparative data in laparoscopic inguinal hernia repair are limited, particularly with regard to patient-reported recovery. We compared the effects of these two blocks on postoperative pain and quality of recovery.

**Methods:**

This prospective observational comparative study included 63 adults (American Society of Anesthesiologists [ASA] I–III) undergoing elective unilateral laparoscopic total extraperitoneal inguinal hernia repair under general anesthesia. At the end of surgery, patients were allocated to receive either an M-TAPA block (*n* = 32) or a lateral TAP block (*n* = 31) within a multimodal analgesia regimen; both blocks are used routinely for postoperative analgesia in our department, and the block given to each patient was chosen by the attending anesthesiologist as part of routine clinical care, without a predefined selection rule and independently of patient characteristics; no randomization was performed. Both blocks were performed bilaterally with 20 mL of 0.25% bupivacaine per side under ultrasound guidance. The co-primary outcomes were the resting Numeric Rating Scale (NRS) pain score at 24 h — the time point on which the sample size calculation was based — and quality of recovery assessed with the Quality of Recovery–15 (QoR-15) score at baseline, 24 h, and postoperative day 15. Resting NRS at earlier time points (PACU arrival and 15 and 30 min) and at 2, 4, 6, and 12 h, movement NRS at all time points, 24-h tramadol consumption, rescue medication use, and postoperative nausea and vomiting (PONV) were secondary, exploratory outcomes.

**Results:**

Baseline characteristics were comparable between groups. Neither co-primary outcome differed significantly between groups: resting NRS at 24 h (median 0 [0–1] in both groups; *p* = 0.566) and QoR-15 scores at any time point (all *p* > 0.05), which returned to baseline by day 15. In secondary, exploratory analyses, resting NRS at 15 and 30 min in the PACU was significantly lower in the lateral TAP group (*p* = 0.010 and *p* = 0.013, respectively); NRS did not differ between groups at any other secondary time point. Cumulative 24-h tramadol consumption did not differ significantly between groups (median 50 mg [30–90] in the TAP group vs 107.5 mg [30–147.5] in the M-TAPA group; *p* = 0.090). PONV incidence and rescue medication use did not differ. No block-related complications occurred.

**Conclusions:**

On the prespecified co-primary outcomes, ultrasound-guided lateral TAP and M-TAPA blocks provided comparable postoperative analgesia at 24 h and comparable quality of recovery. In secondary, exploratory analyses, the lateral TAP block produced lower NRS scores during the early PACU period, possibly because its injection site lies closer to the surgical area; this finding should be regarded as hypothesis-generating and requires confirmation in adequately powered trials. Both techniques can be considered safe alternatives within a multimodal analgesia regimen.

**Clinical trial number:**

ClinicalTrials.gov, NCT06753240; retrospectively registered on 22 December 2024, after completion of patient enrollment.

## Background

Laparoscopic surgery is widely used for inguinal hernia repair and may be associated with moderate to severe postoperative pain [1]. Adequate perioperative pain control is important for early mobilization, reduction of postoperative side effects, shorter hospital stay, improved patient satisfaction, and prevention of chronic postsurgical pain [1]. Recovery from anesthesia and surgery is influenced by multiple factors, including preoperative status, type of surgery, postoperative pain intensity, and analgesic strategy. Patient-centered tools such as the Quality of Recovery–15 (QoR-15) questionnaire have been developed and recommended for clinical research evaluating postoperative comfort and pain [2, 3]. The QoR-15 contains 15 items across five domains—pain, physical comfort, physical independence, psychological support, and emotional state—yielding a total score from 0 to 150, with higher scores indicating better recovery quality [3].

Ultrasound-guided abdominal plane blocks have become an integral component of multimodal analgesia because of their ease of application, prolonged postoperative analgesia, and opioid-sparing effect [4]. Several regional techniques can be used to control pain after laparoscopic inguinal hernia repair, including the transversus abdominis plane (TAP) block and the modified thoracoabdominal nerve block through perichondrial approach (M-TAPA) [4, 5]. The TAP block deposits local anesthetic in the neurofascial plane between the internal oblique and transversus abdominis muscles, producing dermatomal sensory blockade between T6 and L1; it can be performed via lateral, subcostal, or posterior approaches [5, 6]. The M-TAPA block is a more recently described technique in which local anesthetic is injected under the costal cartilage between the transversus abdominis and internal oblique muscles, providing analgesia of the anterior and lateral abdominal wall over a wider dermatomal range (T4–L1) [7, 8].

Although the analgesic effects of TAP and M-TAPA blocks have been investigated separately in various abdominal procedures using conventional pain scores, comparative evidence in laparoscopic inguinal hernia repair—particularly regarding patient-reported quality of recovery—remains limited. Both techniques are routinely used in our department as components of multimodal analgesia. The M-TAPA block was originally introduced for analgesia after upper abdominal surgery, where its subcostal injection site lies close to the surgical field. Dermatomal studies nevertheless suggest that local anesthetic can spread caudally as far as the T12–L1 dermatomes [7, 8], raising the possibility that the block may also be applicable to lower abdominal procedures such as inguinal hernia repair. Whether this caudal spread is sufficient to provide analgesia comparable to that of a lateral TAP block, whose injection site lies directly over the surgical field, has not been established.

The primary aim of this prospective observational comparative study was to compare the effects of the lateral TAP block and the M-TAPA block on two co-primary outcomes — postoperative pain, assessed with the resting NRS score at 24 h, and quality of recovery (QoR-15 score) — in patients undergoing laparoscopic inguinal hernia repair. The secondary aim was to contribute to the identification of a standard analgesic approach that reduces postoperative opioid consumption and nausea and vomiting and improves recovery quality, and to describe, in secondary exploratory analyses, the time-course of postoperative pain at earlier and later time points.

## Methods

### Study design and ethical approval

This single-center prospective observational comparative study was conducted at the Department of Anesthesiology and Reanimation, Fatih Sultan Mehmet Health Application and Research Center, University of Health Sciences (Istanbul, Türkiye), between January 10, 2024, and October 17, 2024. The protocol was approved by the institutional ethics committee (FSM EAH-KAEK approval no. 14.12.2023/23) and registered at ClinicalTrials.gov (NCT06753240). The trial was registered retrospectively, after patient enrollment had been completed. The study was conducted in accordance with the Declaration of Helsinki, and written informed consent was obtained from all participants on the day before surgery. Reporting follows the applicable items of the Strengthening the Reporting of Observational Studies in Epidemiology (STROBE) guideline. Both the M-TAPA and lateral TAP blocks are used routinely for postoperative analgesia after abdominal surgery in our department. The block administered to each patient was chosen by the attending anesthesiologist as part of routine clinical care, without a predefined selection rule and independently of patient characteristics; no randomization sequence, allocation concealment, or systematic assignment rule (such as alternation or allocation by date or record number) was used. The study was therefore designed and registered as a prospective observational comparative study (ClinicalTrials.gov NCT06753240). Blinding was not feasible, because the two techniques differ in needle insertion site and sonoanatomic landmarks and because the same anesthesia team performed the blocks and recorded the postoperative assessments; neither patients nor outcome assessors could therefore be masked to the block received.

### Participants

Adults aged 18–65 years with a body mass index (BMI) below 35 kg/m2, ASA physical status I–III, scheduled for elective unilateral laparoscopic total extraperitoneal (TEP) inguinal hernia repair under general anesthesia, were eligible. Exclusion criteria were refusal to participate; recurrent or bilateral hernia; BMI ≥ 35 kg/m2; local infection or hematoma at the block site; coagulopathy; known local anesthetic allergy or history of local anesthetic systemic toxicity (LAST); decompensated or end-stage hepatic, renal, hematologic, cardiac, or respiratory disease; neurologic or psychiatric disorders including dementia or Alzheimer's disease; chronic analgesic use (applied on the basis of the attending anesthesiologist's clinical judgment at the preoperative visit, as regular ongoing use of any analgesic; no fixed duration, drug class, or dose threshold was prespecified); chronic alcohol use or substance dependence; inability to communicate in Turkish; emergency surgery; and development of subcutaneous emphysema due to CO₂ insufflation.

### Anesthesia management

All patients underwent standard monitoring (electrocardiography, non-invasive blood pressure, peripheral oxygen saturation [SpO₂], body temperature, and bispectral index [BIS]). Premedication was intravenous midazolam 0.02 mg/kg, followed by preoxygenation with 100% oxygen via face mask for three minutes. Anesthesia was induced with intravenous propofol 2–2.5 mg/kg, fentanyl 2 µg/kg, and rocuronium 0.6 mg/kg, and the trachea was intubated after adequate neuromuscular blockade. Volume-controlled ventilation used a tidal volume of 6–8 mL/kg, targeting an end-tidal carbon dioxide (EtCO₂) of 35–40 mmHg. Anesthesia was maintained with sevoflurane 1.5–2% in 50% oxygen/air at a fresh gas flow of 2 L/min and remifentanil 0.05–0.5 µg/kg/min, titrated to keep BIS between 40 and 60. Normothermia was preserved with a forced-air warming device. Twenty minutes before the end of surgery, all patients received intravenous paracetamol 1 g, tramadol 1 mg/kg, and ondansetron 4 mg as standard postoperative analgesia and PONV prophylaxis.

### Block techniques

After completion of surgery and before emergence, with the patient supine and under aseptic conditions, bilateral abdominal plane blocks were performed using a high-frequency linear ultrasound probe (Esaote S.p.A., Genoa, Italy) and a 21G, 100 mm block needle (Stimuplex® A; B. Braun, Bethlehem, PA, USA). All blocks were performed by anesthetists experienced in ultrasound-guided regional techniques.

**Fig. 1:**
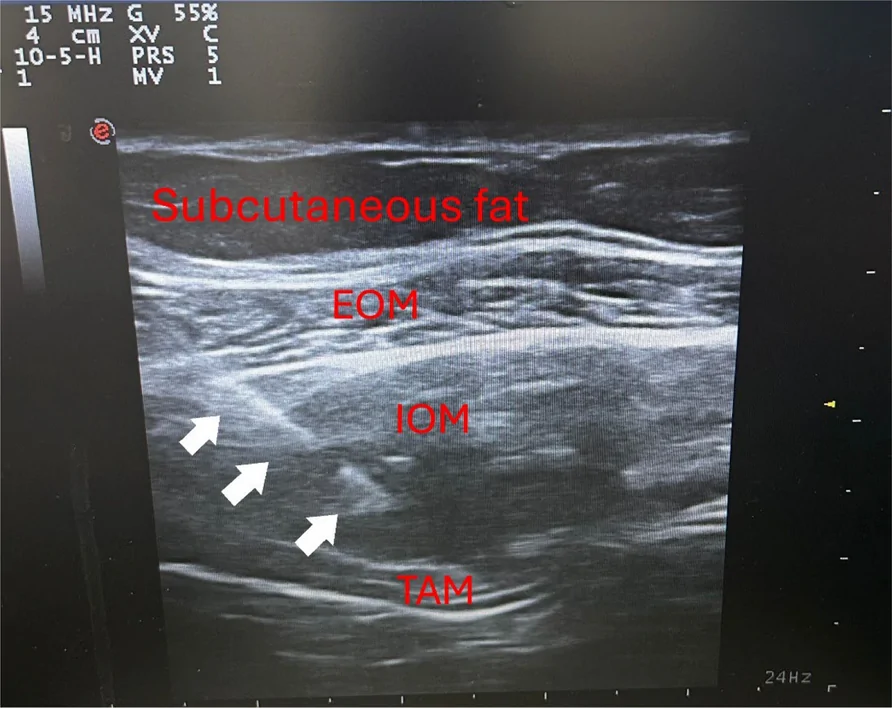
Ultrasound image of the lateral transversus abdominis plane (TAP) block. White arrowheads indicate the needle trajectory. EOM, external oblique muscle; IOM, internal oblique muscle; TAM, transversus abdominis muscle

For the lateral TAP block, the probe was placed transversely between the iliac crest and the costal margin at the mid-axillary line. After identifying the skin and subcutaneous tissue, external oblique, internal oblique, transversus abdominis muscles, and peritoneum, the needle tip was advanced in-plane into the plane between the internal oblique and transversus abdominis muscles and confirmed with hydrodissection of a few milliliters of saline (Fig. 1). After negative aspiration, 20 mL of 0.25% bupivacaine was injected under real-time visualization. The procedure was repeated contralaterally.

**Fig. 2:**
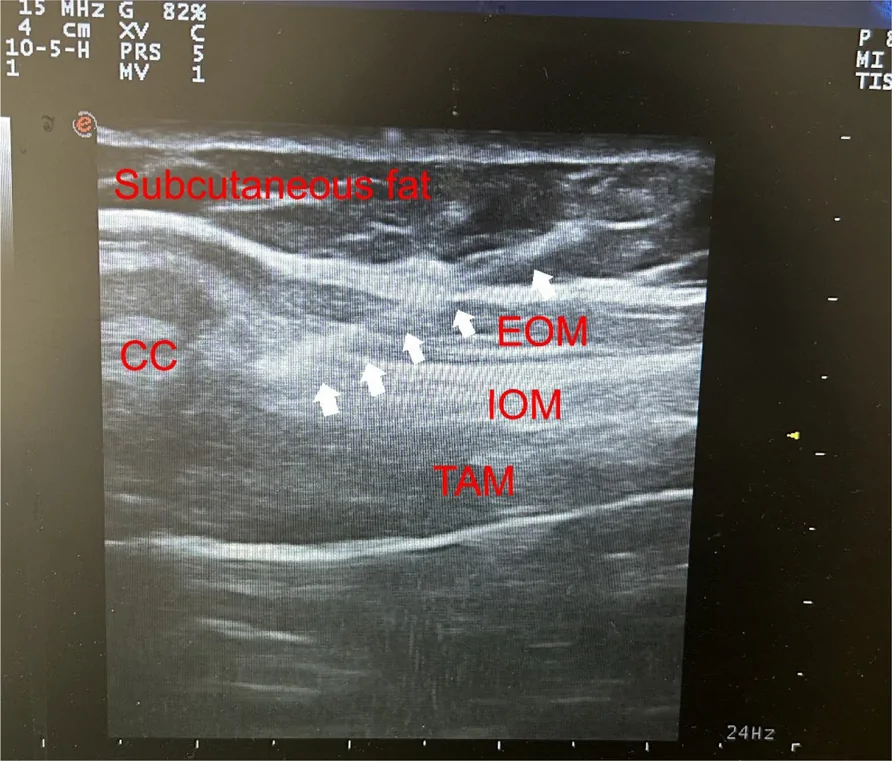
Ultrasound image of the modified thoracoabdominal nerve block through perichondrial approach (M-TAPA). White arrowheads indicate the needle trajectory. CC, costal cartilage; EOM, external oblique muscle; IOM, internal oblique muscle; TAM, transversus abdominis muscle

For the M-TAPA block, the costochondral angle was identified at the mid-clavicular line, and the linear probe was positioned in a sagittal orientation at the lateral edge of the tenth costal cartilage with a deep angle to visualize its inferior surface. Using an in-plane technique in the cephalad direction, the needle tip was advanced beneath the tenth costal cartilage into the plane between the internal oblique and transversus abdominis muscles, and the position was confirmed with a small saline injection (Fig. 2). After negative aspiration, 20 mL of 0.25% bupivacaine was injected. The procedure was performed bilaterally. At the end of the procedure, anesthetic agents were discontinued, sugammadex 2 mg/kg was administered to reverse neuromuscular blockade, and patients were transferred to the post-anesthesia care unit (PACU) once consciousness, muscle strength, and hemodynamic stability were restored.

### Postoperative management and outcomes

In the PACU, all patients received patient-controlled analgesia with tramadol (10 mg bolus, 15-min lockout, no background infusion). Pain was assessed using a 10-point Numeric Rating Scale (NRS; 0 = no pain, 10 = worst imaginable pain). Patients with an NRS score ≥ 4 within the first 20 min received intravenous dexketoprofen 50 mg as rescue analgesia, repeated every 12 h if needed. Intravenous paracetamol 1 g every six hours was continued on the ward; if resting NRS remained ≥ 4, the tramadol bolus was increased to 15 mg. PONV was assessed at each follow-up; ondansetron 4 mg was the first-line antiemetic, followed by intravenous dexamethasone 4 mg if symptoms persisted. Patients were transferred to the ward when the Aldrete score reached 9 and resting NRS was below 4.

The co-primary outcomes were postoperative pain intensity, assessed with the resting NRS at 24 h, and quality of recovery, assessed with the QoR-15 score measured preoperatively, at 24 h, and on postoperative day 15. Secondary, exploratory outcomes were resting NRS at PACU arrival and at 15 and 30 min and at 2, 4, 6, and 12 h; movement NRS at 2, 4, 6, 12, and 24 h; cumulative 24-h tramadol consumption; rescue analgesic and antiemetic requirements; presence of PONV (yes/no); and block-related complications including LAST. The validated Turkish version of the QoR-15 was used [2]; the day-15 assessment was conducted by telephone interview.

### Sample size and statistical analysis

Sample size was based on the co-primary pain outcome, the 24-h resting NRS, using the reference value reported by Vaghela et al. [9] (24-h postoperative NRS of 3.88 ± 1.45 after inguinal hernia surgery with a TAP block). A 30% between-group difference was assumed, corresponding to approximately 1.2 NRS points; this magnitude was chosen because it approximates the minimal clinically important difference (MCID) of 1–2 points reported for acute postoperative pain [10, 11], so that smaller differences would not be considered clinically meaningful. With an alpha of 0.05 and 80% power, a minimum of 48 patients was required; 63 were included (M-TAPA, n = 32; lateral TAP, n = 31). No comparable prior data were available on the expected between-group difference or variance in QoR-15 scores for this specific surgical population at the time of protocol design, so the sample size calculation could not be based on the co-primary QoR-15 outcome; analyses of that outcome should therefore be regarded as having limited statistical power. All other postoperative NRS time points (PACU arrival and 15 and 30 min; 2, 4, 6, and 12 h; movement NRS at all time points) were secondary, exploratory analyses of the time-course of pain and were not used for sample size planning. Data were analyzed with SPSS version 27.0 (IBM Corp., Armonk, NY, USA). Distribution normality was assessed with the Kolmogorov–Smirnov and Shapiro–Wilk tests. Normally distributed continuous variables were compared with the independent-samples t-test and are presented as mean ± standard deviation; non-normally distributed variables were compared with the Mann–Whitney U test and are presented as median (interquartile range, Q1–Q3). Categorical variables were compared with the chi-square or Fisher's exact test and are presented as frequency (percentage). For the co-primary NRS outcome, the co-primary QoR-15 outcome, the secondary NRS time points, and cumulative tramadol consumption, effect sizes are reported as the rank-biserial correlation (r), derived from the standardized test statistic of the Mann–Whitney U test as r = z/√N, together with 95% confidence intervals obtained by Fisher's z-transformation. A two-tailed p < 0.05 was considered statistically significant for the co-primary outcomes. Because the secondary NRS time points were exploratory and were not the basis of the sample size calculation, the corresponding p-values were not adjusted for multiplicity and these comparisons should be interpreted as hypothesis-generating rather than confirmatory.

## Results

### Patient characteristics

**Fig. 3:**
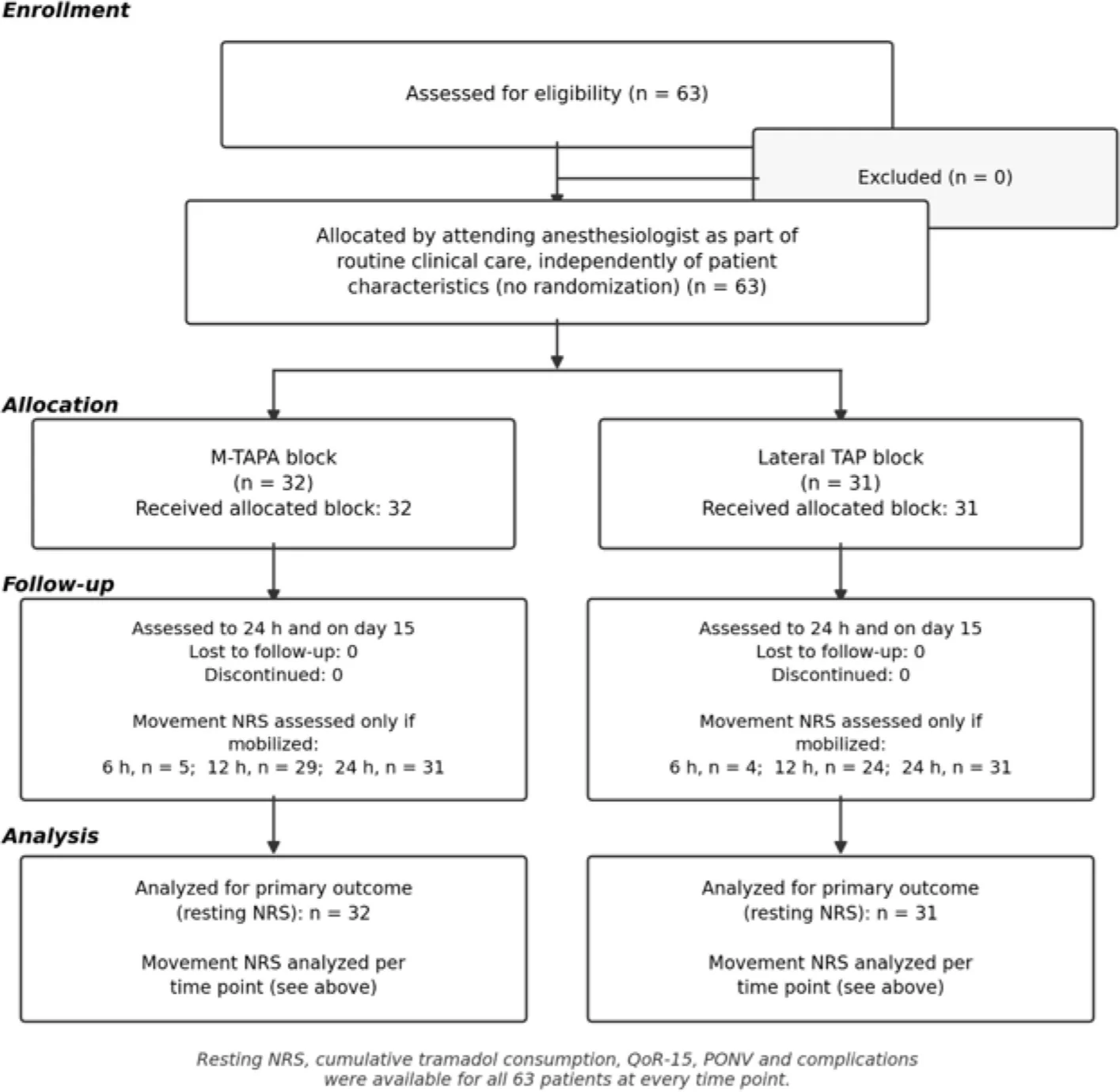
Patient flow through the study. Block allocation was made by the attending anesthesiologist as part of routine clinical care, without a predefined selection rule and independently of patient characteristics; no randomization sequence or allocation concealment was used. Movement NRS could be recorded only in patients who had mobilized at the relevant time point. NRS, Numeric Rating Scale

A total of 63 patients (58 men and 5 women; mean age 51.7 ± 12.6 years) were included; 32 received the M-TAPA block and 31 the lateral TAP block. Demographic data, ASA classification, comorbidities, and operative times were similar between groups (Table 1). No patient was excluded after enrollment, and there were no protocol deviations (Fig. 3).

**Table 1:** Demographic and clinical characteristics of the study population

Variable	M-TAPA (*n* = 32)	TAP (*n* = 31)	*p*-value
Age (years), mean ± SD	51.0 ± 12.8	52.5 ± 13.0	0.636
Sex, male/female, n (%)	30 (93.8)/2 (6.3)	28 (90.3)/3 (9.7)	0.615
Height (cm), median (IQR)	174 (168–178.75)	172 (169–178)	0.782
Weight (kg), mean ± SD	76.2 ± 11.2	77.1 ± 14.9	0.792
BMI (kg/m^2^), mean ± SD	25.4 ± 3.2	25.6 ± 4.3	0.854
ASA I/II/III, n (%)	12 (37.5)/14 (43.8)/6 (18.8)	11 (35.5)/14 (45.2)/6 (19.4)	0.986
Cardiovascular disease, n (%)	4 (12.5)	3 (9.7)	0.722
Hypertension, n (%)	6 (18.8)	7 (22.6)	0.707
Diabetes mellitus, n (%)	4 (12.5)	2 (6.5)	0.414
Active smoker, n (%)	8 (25.0)	12 (38.7)	0.243
Operative time (min), mean ± SD	56.8 ± 18.2	52.1 ± 19.4	0.326
Anesthesia time (min), mean ± SD	73.1 ± 18.1	69.3 ± 20.3	0.430

Data are mean ± standard deviation, median (interquartile range), or n (%). Age, weight, BMI, operation time, and anesthesia time were compared with the independent-samples t-test; height with the Mann–Whitney U test; categorical variables with the chi-square or Fisher's exact test

*ASA* American Society of Anesthesiologists, *BMI* Body mass index, *IQR* Interquartile range, *SD* Standard deviation, *M-TAPA* Modified thoracoabdominal nerve block through perichondrial approach, *TAP* Transversus abdominis plane

### Postoperative pain scores

On the co-primary outcome, resting NRS at 24 h did not differ between groups (median 0 [0–1] in both groups; *p* = 0.566, *r* = 0.07, 95% CI − 0.18 to 0.31). In secondary, exploratory analyses, resting NRS at 15 and 30 min in the PACU was significantly lower in the lateral TAP group than in the M-TAPA group (median 0 [0–2] vs 2 [0–3], *p* = 0.010, *r* = 0.32, 95% CI 0.08 to 0.53; and median 0 [0–2] vs 2 [0.25–2.75], *p* = 0.013, *r* = 0.31, 95% CI 0.07 to 0.52, respectively). The absolute median difference at both time points was 2 points, although the corresponding difference in mean scores was approximately 1 point. Resting NRS at PACU arrival and at 2, 4, 6, and 12 h did not differ between groups. Movement NRS was assessed only in patients who were able to mobilize at the relevant time point, and did not differ between groups at 6 h (*n* = 5 and 4 patients, respectively), 12 h (*n* = 29 and 24), or 24 h (*n* = 31 and 31); the 6-h comparison is based on too few patients to be interpreted (Table 2).

**Table 2:** Resting and movement NRS scores in the postoperative period

Time point	M-TAPA (*n* = 32)	TAP (*n* = 31)	*p*-value	Effect size, r (95% CI)
*Resting NRS*				
PACU arrival	2 (0–2.75)	0 (0–2)	0.075	0.22 (− 0.02 to 0.45)
PACU 15 min	2 (0–3)	0 (0–2)	0.010*	0.32 (0.08 to 0.53)
PACU 30 min	2 (0.25–2.75)	0 (0–2)	0.013*	0.31 (0.07 to 0.52)
Postoperative 2 h	1 (1–2)	1 (0–2)	0.260	0.14 (− 0.11 to 0.38)
Postoperative 4 h	1 (0–2)	1 (0–2)	0.406	0.10 (− 0.15 to 0.34)
Postoperative 6 h	0.5 (0–2)	1 (0–1)	0.929	0.01 (− 0.24 to 0.26)
Postoperative 12 h	0 (0–1)	0 (0–1)	0.431	0.10 (− 0.15 to 0.34)
Postoperative 24 h‡	0 (0–1)	0 (0–1)	0.566	0.07 (− 0.18 to 0.31)
*Movement NRS*				
Postoperative 6 h (n = 5/4)†	0 (0–2.5)	0.5 (0–1)	0.787	—
Postoperative 12 h (n = 29/24)†	2 (0–3)	2 (1–3)	0.509	—
Postoperative 24 h (n = 31/31)†	2 (0–3)	1 (0–2)	0.606	—

Data are median (interquartile range, Q1–Q3); Mann–Whitney U test. Effect size is the rank-biserial correlation (r) with its 95% confidence interval; positive values indicate higher pain scores in the M-TAPA group

*CI* Confidence interval, *NRS* Numeric Rating Scale, *PACU* Post-anesthesia care unit, *M-TAPA* Modified thoracoabdominal nerve block through perichondrial approach, *TAP* Transversus abdominis plane

‡Co-primary outcome (24-h resting NRS); the sample size calculation was based on this time point. All other resting NRS time points and all movement NRS time points are secondary, exploratory analyses. †Movement NRS could be assessed only in patients who were able to mobilize at that time point; the number of patients assessed in each group is given as (M-TAPA/TAP). Because only 5 and 4 patients, respectively, were mobilized at 6 h, the 6-h movement NRS is reported for completeness and should not be interpreted

*Statistically significant (*p* < 0.05)

### Tramadol consumption and rescue medication

Cumulative tramadol consumption did not differ significantly between groups at any time point. Median consumption was identical or closely similar in the two groups during the first 4 postoperative hours, whereas from 6 h onward it was numerically lower in the lateral TAP group. The 24-h cumulative consumption was 50 mg (30–90) in the TAP group versus 107.5 mg (30–147.5) in the M-TAPA group, a difference that did not reach statistical significance (*p* = 0.090, *r* = 0.21, 95% CI − 0.04 to 0.44) (Table 3). In the M-TAPA group, tramadol consumption began to increase from the sixth postoperative hour. Three patients (9.4%) in the M-TAPA group required rescue dexketoprofen in the PACU, whereas no TAP patient required rescue analgesia (*p* = 0.238). No patient in either group required rescue analgesia on the ward. One patient in the M-TAPA group had a resting NRS ≥ 4 on the ward and received the increased 15 mg tramadol bolus; no other patient required a dose adjustment.

**Table 3:** Cumulative tramadol consumption (mg) over the first 24 postoperative hours

Time point	M-TAPA (n = 32)	TAP (n = 31)	*p*-value	Effect size, r (95% CI)
PACU 15 min	0 (0–0)	0 (0–0)	0.670	0.05 (− 0.20 to 0.30)
PACU 30 min	0 (0–10)	0 (0–10)	0.711	0.05 (− 0.20 to 0.29)
Postoperative 2 h	10 (10–30)	10 (10–20)	0.451	0.09 (− 0.16 to 0.33)
Postoperative 4 h	25 (10–50)	20 (10–30)	0.289	0.13 (− 0.12 to 0.37)
Postoperative 6 h	30 (10–70)	20 (10–40)	0.194	0.16 (− 0.09 to 0.40)
Postoperative 12 h	50 (22.5–117.5)	30 (20–50)	0.137	0.19 (− 0.06 to 0.42)
Postoperative 24 h	107.5 (30–147.5)	50 (30–90)	0.090	0.21 (− 0.04 to 0.44)

Data are median (interquartile range, Q1–Q3); Mann–Whitney U test. Values are cumulative. Effect size is the rank-biserial correlation (r) with its 95% confidence interval; positive values indicate higher tramadol consumption in the M-TAPA group

*CI* Confidence interval, *PACU* Post-anesthesia care unit, *M-TAPA* Modified thoracoabdominal nerve block through perichondrial approach, *TAP* Transversus abdominis plane

### Postoperative nausea and vomiting

PONV was infrequent in both groups. In the PACU, no TAP patient experienced PONV, whereas one M-TAPA patient (3.1%) reported nausea (*p* = 1.000). At 12 and 24 h, PONV was numerically more frequent in the M-TAPA group (3/32 [9.4%] and 2/32 [6.3%]) than in the TAP group (1/31 [3.2%] and 0/31), without statistical significance. Antiemetic use during follow-up was similar between groups (all *p* > 0.05).

### Quality of recovery

On the co-primary quality-of-recovery outcome, QoR-15 scores did not differ between groups at any time point (Mann–Whitney U test throughout, as this variable did not meet the normality assumption at any time point). Preoperative scores were similar (median 148 [144.25–150] in the M-TAPA group vs 147 [145–148] in the TAP group; *p* = 0.352, *r* = 0.12, 95% CI − 0.13 to 0.35). At 24 h, scores decreased slightly in both groups (median 143 [139–145] vs 143 [139–146]; *p* = 0.586, *r* = − 0.07, 95% CI − 0.31 to 0.18) and returned to baseline by postoperative day 15 (median 149 [145.5–150] vs 148 [143–150]; *p* = 0.662, *r* = 0.06, 95% CI − 0.20 to 0.30) (Table 4).

**Table 4:** QoR-15 scores at baseline, 24 h, and 15 days postoperatively

Time point	M-TAPA (*n* = 32)	TAP (*n* = 31)	*p*-value	Effect size, r (95% CI)
Preoperative, median (IQR)	148 (144.25–150)	147 (145–148)	0.352	0.12 (− 0.13 to 0.35)
Postoperative 24 h, median (IQR)	143 (139–145)	143 (139–146)	0.586	− 0.07 (− 0.31 to 0.18)
Postoperative day 15, median (IQR)	149 (145.5–150)	148 (143–150)	0.662	0.06 (− 0.20 to 0.30)

QoR-15 is a co-primary outcome, assessed at all three time points shown; because this variable did not meet the normality assumption (Kolmogorov–Smirnov/Shapiro–Wilk) at any time point, all three comparisons were made with the Mann–Whitney U test and data are presented as median (interquartile range). Effect size is the rank-biserial correlation (r) with its 95% confidence interval; positive values indicate a higher (better) QoR-15 score in the M-TAPA group

*IQR* Interquartile range, *QoR-15* Quality of Recovery–15, *CI* Confidence interval, *M-TAPA* Modified thoracoabdominal nerve block through perichondrial approach, *TAP* Transversus abdominis plane

### Block-related complications

No block-related complications, including peritoneal puncture, hematoma, infection, or local anesthetic systemic toxicity, occurred in either group during the perioperative period or follow-up.

## Discussion

In this prospective observational comparative study of two ultrasound-guided abdominal plane blocks in patients undergoing laparoscopic inguinal hernia repair, the two prespecified co-primary outcomes — resting NRS at 24 h and QoR-15 score — did not differ between the lateral TAP and M-TAPA blocks, indicating comparable analgesia and quality of recovery on the confirmatory comparisons for which the study was powered. In secondary, exploratory analyses, the lateral TAP block produced significantly lower resting NRS scores than the M-TAPA block at 15 and 30 min in the PACU, whereas pain scores, opioid consumption, and PONV were similar between groups at all other time points.

The thoracoabdominal nerves are the anterior and posterior branches of the thoracic spinal nerves; they emerge from the intercostal spaces and run within the plane between the internal oblique and transversus abdominis muscles, with sensory and motor innervation of the abdominal wall supplied by the anterior primary rami of T7–T12 [12]. Balaban et al. described the thoracoabdominal nerve block through perichondrial approach (TAPA), in which local anesthetic is administered to both the upper and lower surfaces of the costal cartilage at the ninth and tenth costal levels, producing sensory blockade in the T5–T12 dermatomes [13]. Tulgar et al. subsequently described the modified TAPA (M-TAPA), which limits injection to the lower surface of the costal cartilage, thereby blocking the anterior and lateral branches of the T4/T5–T12/L1 thoracoabdominal nerves [8]. In a cadaveric study, Çiftçi et al. showed that methylene blue spread over the transversus abdominis muscle with both approaches, with greater spread observed with M-TAPA, and concluded that the efficacy of fascial plane blocks is volume-dependent [7, 12].

Several studies have demonstrated the analgesic efficacy of M-TAPA in upper abdominal surgery. Avcı et al. [14] and Bilge et al. [15] reported lower NRS scores and reduced opioid consumption with M-TAPA compared with controls after laparoscopic cholecystectomy. Güngör et al. [16] showed that bilateral M-TAPA with 40 mL of 0.25% bupivacaine produced lower NRS scores in the first 4 h at rest and 16 h on movement compared with surgical site infiltration. M-TAPA has also been compared with other plane blocks: Bilge et al. found lower 24-h opioid consumption with M-TAPA than with the oblique subcostal TAP (OSTAP) block [17], whereas Cho et al. found no significant difference in pain, rescue opioid use, or satisfaction between M-TAPA and subcostal TAP block [18]. In laparoscopic sleeve gastrectomy, Turunç et al. reported no difference between bilateral M-TAPA and external oblique intercostal block [19]. In a recent randomized trial, Teke et al. compared M-TAPA with the OSTAP block in laparoscopic sleeve gastrectomy and found significantly lower tramadol consumption with M-TAPA at the sixth postoperative hour (*p* = 0.009), with a comparable trend in cumulative 24-h consumption [20].

Evidence specific to lower abdominal surgery, and particularly to laparoscopic inguinal hernia repair, is more limited. Bilgili et al. compared M-TAPA with wound infiltration in major abdominal surgery and reported significantly lower NRS scores and morphine consumption beyond the second postoperative hour with M-TAPA [21]. Rauf et al. described a case in which bilateral M-TAPA provided adequate analgesia without opioid requirement after bilateral laparoscopic inguinal hernia repair [22]. In the only randomized study to date specifically addressing M-TAPA in laparoscopic inguinal hernia repair, Alver et al. found that M-TAPA reduced resting and movement NRS scores during the first 8 h, rescue analgesic requirement, and side-effect incidence compared with surgical site infiltration [1]. Atsumi et al. reported no significant difference between M-TAPA and wound infiltration in 24-h analgesic requirement after gynecological laparoscopic surgery [23], while Suzuka et al. found no superiority of M-TAPA over OSTAP regarding NRS, opioid consumption, or quality of recovery in laparoscopic hysterectomy [24].

The analgesic efficacy of the lateral TAP block is well established; the lateral approach blocks the anterior cutaneous branches of T10–T12 [25, 26]. In open inguinal hernia repair, Theodoraki et al. showed that lateral TAP with 20 mL of 0.75% ropivacaine reduced 24-h resting and movement NRS, morphine consumption, and intraoperative remifentanil requirement compared with placebo, although it did not reduce chronic pain [27]. Okur et al. reported lower 24-h NRS scores with TAP than with ilioinguinal/iliohypogastric block in unilateral inguinal hernia repair [28]. In laparoscopic inguinal hernia repair specifically, Sivapurapu et al. demonstrated lower 12-h movement NRS, lower 24-h resting NRS, and prolonged time to first rescue analgesic with bilateral midaxillary TAP versus control [29], whereas Asokan et al. found no difference between bilateral TAP and port-site infiltration [30].

The dermatomal behavior of the M-TAPA block offers a plausible anatomical explanation for our findings. In a cadaveric investigation, Çiftçi et al. found that dye injected beneath the costal cartilage spread over the thoracoabdominal nerves between T4 and T12, with more extensive spread over the transversus abdominis muscle after M-TAPA than after TAPA [12]. In their clinical case series, sensory blockade was mapped to the T6–T12 dermatomes [7], and Tulgar et al. described coverage of the T4/T5–T12/L1 nerves [8]. Across these reports the blockade is concentrated in the mid and upper abdominal dermatomes, and involvement of T12 and L1 is inconsistent, being reported at the caudal margin of the distribution rather than as a reliable finding. The surgical field of laparoscopic inguinal hernia repair corresponds to the T12–L1 dermatomes; the M-TAPA injection site is therefore several segments cephalad to the site of nociceptive input, and adequate analgesia depends on caudal spread that may vary between patients. By contrast, the lateral TAP block deposits local anesthetic directly within the plane supplying T10–T12, immediately adjacent to the surgical field. This anatomical asymmetry is consistent with the lower early pain scores observed in the lateral TAP group and with the fact that the difference disappeared once systemic multimodal analgesia had reached steady state. It also implies that the analgesic efficacy reported for M-TAPA after upper abdominal surgery, and its benefit over surgical infiltration in laparoscopic inguinal hernia repair [1], should not be extrapolated to a direct comparison with a block sited over the inguinal region.

Although identified in a secondary, exploratory analysis rather than the prespecified co-primary comparison, the clinical relevance of the early difference in pain scores deserves careful interpretation. At 15 and 30 min in the PACU the difference in median resting NRS was 2 points, whereas the difference in mean scores was approximately 1 point, with moderate effect sizes (*r* = 0.32 and *r* = 0.31, respectively). The minimal clinically important difference for acute postoperative pain is generally reported as 1 to 2 points on an 11-point scale, corresponding to about 10 mm on a 100 mm visual analog scale [10, 11]. The observed difference therefore lies at the lower boundary of what patients are likely to perceive as a meaningful change, and the lower limits of the confidence intervals for the effect sizes (0.08 and 0.07) are compatible with a difference of little clinical importance. In addition, resting NRS scores were low in both groups at every time point (medians ≤ 2), rescue analgesia in the PACU was required by only three patients, and no patient in either group required rescue analgesia on the ward. Taken together, these findings suggest that although the lateral TAP block conferred a statistically significant early advantage, both blocks provided pain control within a range that would generally be considered acceptable, and the early difference should not by itself be regarded as establishing clinical superiority.

In our study, patients receiving lateral TAP had significantly lower NRS scores during the early postoperative period (PACU) than those receiving M-TAPA, while ward NRS scores at rest and on movement were similar. This early difference may reflect the closer anatomical proximity of the lateral TAP injection site to the lower-abdominal surgical area and earlier diffusion of local anesthetic toward the inguinal region. Although not statistically significant, cumulative tramadol consumption was similar during the first postoperative hours and became numerically lower in the TAP group from the sixth hour onward, with the largest difference at 24 h (median 50 mg in the TAP group vs 107.5 mg in the M-TAPA group); the upward trend in the M-TAPA group from the sixth hour may reflect earlier waning of the analgesic effect at the surgical site. Notably, the direction of this difference is opposite to that reported by Teke et al. in laparoscopic sleeve gastrectomy, where M-TAPA was associated with lower tramadol consumption than OSTAP [20]. This apparent discrepancy is consistent with an anatomical rationale: in upper-abdominal bariatric surgery the perichondrial M-TAPA injection lies close to the surgical field, whereas in lower-abdominal hernia repair the lateral TAP injection site is closer to the operative area. No patient required rescue analgesia on the ward, suggesting that both techniques produced clinically adequate analgesia within a multimodal regimen during the first 24 h.

Quality of recovery is increasingly recognized as a patient-centered outcome that captures aspects of postoperative care not reflected by traditional metrics such as length of stay or complication rates [3, 31]. The QoR-15 has been validated as a global measure of postoperative recovery across diverse surgical populations [3]. Bilge et al. reported higher 24-h QoR-40 scores with M-TAPA than control in laparoscopic cholecystectomy [15]. Turunç et al. and Suzuka et al. found no significant difference in QoR-15 scores between M-TAPA and other plane blocks in laparoscopic sleeve gastrectomy and hysterectomy, respectively [19, 24]. Park et al. demonstrated higher third-day QoR-40 scores with TAP versus control in laparoscopic nephrectomy [32], whereas Geng et al. found no difference in 24-h QoR-40 scores between TAP and control in open gynecological surgery [33]. Alver et al. reported higher QoR-40 scores with M-TAPA than wound infiltration after laparoscopic inguinal hernia repair [1]. In our study, QoR-15 scores were similar between groups at all time points and returned to baseline by day 15, indicating comparable contributions to recovery quality and complete recovery to preoperative status within two weeks.

PONV is among the most common complications of general anesthesia and may delay recovery [34]. Multimodal analgesia incorporating fascial plane blocks reduces opioid consumption and may decrease PONV [34]. In our cohort, PONV incidence did not differ significantly between groups; however, a non-significant trend toward higher PONV after the 12th hour in the M-TAPA group paralleled the higher tramadol consumption in that group, consistent with the established association between opioid use and PONV. No patient developed LAST or other block-related complications, supporting the safety of both ultrasound-guided techniques when performed by experienced anesthetists.

### Limitations

This study has several limitations. First, the study was observational and non-randomized: the block given to each patient was chosen by the attending anesthesiologist as part of routine clinical care, without a predefined selection rule and independently of patient characteristics. This carries a substantial risk of selection bias, and residual confounding cannot be excluded, although the groups were comparable in baseline characteristics (Table 1). Blinding of patients and outcome assessors was not feasible, because the two techniques differ in needle insertion site and sonoanatomy and because the same anesthesia team performed the blocks and recorded the postoperative assessments. Second, “chronic analgesic use” was applied as an exclusion criterion on the basis of clinical judgment at the preoperative visit; no fixed duration, drug class, or dose threshold was prespecified, which limits the reproducibility of the eligibility criteria. Third, movement NRS could be assessed only in patients who had mobilized at the relevant time point, so the number of patients assessed varied across time points (6 h, 5 and 4 patients; 12 h, 29 and 24; 24 h, 31 and 31, in the M-TAPA and lateral TAP groups, respectively); the 6-h comparison in particular rests on too few patients to support any inference. Fourth, dermatomal sensory mapping was not performed after either block. Because M-TAPA is a relatively new technique whose reported dermatomal spread varies between studies, and because its efficacy in lower abdominal surgery depends on sufficient caudal spread to the T10–T12 dermatomes, the absence of sensory testing means that successful and comparable sensory blockade could not be confirmed; the observed secondary-analysis differences in early pain scores may therefore partly reflect variability in the extent of block spread rather than an intrinsic difference between the techniques. Fifth, the sample size was modest and was based on the co-primary pain outcome, the 24-h resting NRS; no comparable prior data were available to base a power calculation on the co-primary QoR-15 outcome, and analyses of that outcome should therefore be interpreted with this limitation in mind. All other NRS time points, and the secondary outcomes, were exploratory and were likewise not powered. The 24-h difference in cumulative tramadol consumption (median 107.5 vs 50 mg) did not reach statistical significance, and its effect-size confidence interval was compatible with both no effect and a moderate effect (r = 0.21, 95% CI − 0.04 to 0.44); this observation should therefore be regarded as hypothesis-generating and must not be interpreted as evidence that the techniques are equivalent with respect to opioid consumption. The same reasoning applies to PONV. Sixth, because the secondary NRS time points were exploratory rather than confirmatory, comparisons across these time points were not adjusted for multiplicity; the early PACU difference identified in these secondary analyses was not the prespecified co-primary comparison and warrants confirmation in adequately powered randomized trials designed with that specific hypothesis in mind. Seventh, the sex distribution was heterogeneous, with a male predominance reflecting the epidemiology of inguinal hernia, which may limit generalizability. Finally, only laparoscopic TEP repair was studied, and the findings may not apply to transabdominal preperitoneal (TAPP) or open techniques.

## Conclusions

On the prespecified co-primary outcomes, both ultrasound-guided lateral TAP and M-TAPA blocks, performed bilaterally with 20 mL of 0.25% bupivacaine per side at the end of surgery, provided comparable postoperative analgesia at 24 h and comparable quality of recovery in patients undergoing laparoscopic inguinal hernia repair. In secondary, exploratory analyses, the lateral TAP block produced lower NRS scores in the early postoperative period, most likely because its injection site is anatomically closer to the surgical area; beyond this period, the two techniques produced comparable analgesia, opioid consumption, and PONV incidence. This early difference should be regarded as hypothesis-generating rather than confirmatory. Both blocks were performed without complications and can be considered safe and effective components of a multimodal analgesia regimen for this surgery. Larger, adequately powered randomized trials are warranted to confirm these findings, specifically test the early-analgesia hypothesis, and refine block selection in laparoscopic inguinal hernia repair.

## Data Availability

The datasets generated and analyzed during the current study are available from the corresponding author on reasonable request.

## Abbreviations

ASA: American Society of Anesthesiologists
BIS: Bispectral Index
BMI: Body mass index
CC: Costal cartilage
EOM: External oblique muscle
ESAIC: European Society of Anaesthesiology and Intensive Care
EtCO₂: End-tidal carbon dioxide
IOM: Internal oblique muscle
LAST: Local anesthetic systemic toxicity
M-TAPA: Modified thoracoabdominal nerve block through perichondrial approach
NRS: Numeric Rating Scale
OSTAP: Oblique subcostal transversus abdominis plane
PACU: Post-anesthesia care unit
PONV: Postoperative nausea and vomiting
QoR-15: Quality of Recovery–15
SpO₂: Peripheral oxygen saturation
STROBE: Strengthening the Reporting of Observational Studies in Epidemiology
TAM: Transversus abdominis muscle
TAP: Transversus abdominis plane
TAPA: Thoracoabdominal nerve block through perichondrial approach
TAPP: Transabdominal preperitoneal
TEP: Total extraperitoneal
